# Underutilization of Aspirin Persists in US Ambulatory Care for the Secondary and Primary Prevention of Cardiovascular Disease

**DOI:** 10.1371/journal.pmed.0020353

**Published:** 2005-11-15

**Authors:** Randall S Stafford, Veronica Monti, Jun Ma

**Affiliations:** **1**Program on Prevention Outcomes and Practices, Stanford Prevention Research Center, Stanford University School of Medicine, Stanford, California, United States of America; Radcliffe InfirmaryUnited Kingdom

## Abstract

**Background:**

Despite the proven benefits of aspirin therapy in the primary and secondary prevention of cardiovascular disease (CVD), utilization rates of aspirin remain suboptimal in relation to recommendations. We studied national trends of aspirin use among intermediate- to high-risk patients in the US ambulatory care settings and compared the priority given to aspirin versus statins for CVD risk reduction. We also examined patient and health care provider contributors to the underuse of aspirin.

**Methods and Findings:**

We used the 1993–2003 US National Ambulatory Medical Care Survey and National Hospital Ambulatory Medical Care Survey to estimate aspirin use by cardiovascular risk. Physician-noted cardiovascular diseases defined high risk. Intermediate risk was defined as having diabetes mellitus or multiple major risk factors. The proportion of patient visits in which aspirin was reported increased from 21.7% (95% confidence interval: 18.8%–24.6%) in 1993–1994 to 32.8% (25.2%–40.4%) in 2003 for the high-risk category, 3.5% (2.0%–5.0%) to 11.7% (7.8%–15.7%) for visits by patients diagnosed with diabetes, and 3.6% (2.6%–4.6%) to 16.3% (11.4%–21.2%) for those with multiple CVD risk factors. Beginning in 1997–1998, statins were prioritized over aspirin as prophylactic therapy for reducing CVD risk, and the gaps remained wide through 2003. In addition to elevated CVD risk, greater aspirin use was independently associated with advanced age, male gender, cardiologist care, and care in hospital outpatient departments.

**Conclusion:**

Improvements in use of aspirin in US ambulatory care for reducing risks of CVD were at best modest during the period under study, particularly for secondary prevention, where the strongest evidence and most explicit guidelines exist. Aspirin is more underused than statins despite its more favorable cost-effectiveness. Aggressive and targeted interventions are needed to enhance provider and patient adherence to consensus guidelines for CVD risk reduction.

## Abstract 2

Aspirin is underused compared with statins especially for secondary prevention of cardiovascular disease, despite being more cost-effective.

## Introduction

Cardiovascular disease (CVD), including myocardial infarction and stroke, is the leading cause of morbidity and mortality in the United States. A broad array of randomized trials have demonstrated the benefits of low doses of aspirin (75–325 mg) [1,2] for both the primary [3–7] and secondary prevention [8–11] of CVD. Most trials demonstrate a 15%–40% reduction in cardiovascular events with chronic aspirin use. Aspirin is unequivocally recommended as a secondary prevention strategy in non-contraindicated patients with known CVD [12,13]. As for primary prevention, the American Diabetic Association recommends regular aspirin for men and women with diabetes mellitus (DM) who are older than 40 y or have additional cardiovascular risk factors [14]. In addition, aspirin is indicated for apparently healthy individuals without CVD or DM but otherwise with an increased cardiovascular risk, which is defined as a 3% or greater risk in 5 y by the US Preventive Services Task Force [2] or a 10% or greater risk in 10 y by the American Heart Association [1]. However, the latest results from the Women's Health Study [7] suggest that careful ascertainment of the absolute benefit and risk on a case-by-case basis is essential to deciding on the use of aspirin therapy in men and, even more so, in women who have showed no clinical manifestations of CVD or diabetes.

Despite the proven benefits of aspirin therapy for reducing cardiovascular risk, aspirin use falls considerably short of recommendations. National surveys of the prescribing of cardiac medications found that aspirin use in visits by patients with coronary heart disease (CHD) increased significantly from 5% in 1980 to 32% in 1995, but then remained unchanged or even declined in subsequent years [15,16]. The Third National Health and Nutrition Examination Survey (also called NHANES III) data showed that among patients with DM, only 37% of those with CHD and 13% of those with risk factors for CHD were regular aspirin users [17]. While aspirin underutilization is also present in other countries [18,19], some evidence suggests that the problem is more prominent in the US. For instance, outpatient use of aspirin for secondary prevention ranged from approximately 40% to 90% in many European countries, in comparison to approximately 24% in the US [15,20–23]. Greater aspirin use is associated with middle to older age (55–75 y old), male gender, diagnosis of hyperlipidemia, smoking, having medical insurance, revascularization or coronary angioplasty, and use of other medications [24–28].

Despite ample evidence of aspirin underutilization, research on national trends of outpatient aspirin use by CVD risk category is limited. Using two companion national datasets on ambulatory care in the US, our study tracked changes from 1993–2003 in reported aspirin use by CVD risk status, distinguishing between secondary and primary prevention. Multiple reasons may account for the widespread aspirin underutilization, one being lower priority assigned to aspirin therapy compared to other medications available for CVD risk reduction. To explore this possibility, we examined the priority given to aspirin in comparison to statins. We also examined patient and physician contributors to shortfalls in aspirin use.

## Methods

The Stanford University Institutional Review Board determined that this study was exempt from “human subjects” requirements.

### Data Sources

We obtained annual data 1993–2003 from the National Ambulatory Medical Care Survey (NAMCS) and the Outpatient Department component of the National Hospital Ambulatory Medical Care Survey (NHAMCS). The National Center for Health Statistics provides complete descriptions of both surveys and yearly data at http://www.cdc.gov/nchs/about/major/ahcd/ahcd1.htm. These surveys, particularly NAMCS, have been validated against other data sources [29,30] and utilized in past research on aspirin use for CVD risk reduction [16,25].

In brief, NAMCS captured health-care services provided by private office-based physicians, while NHAMCS captured services offered at hospital outpatient departments. The sampling universe for NAMCS was office-based, patient-care physicians in 15 specialty strata from the master files maintained by the American Medical Association and American Osteopathic Association. The sampling frame for NHAMCS included short-stay (shorter than 30 d) hospitals, or general-specialty (medical or surgical) or children's general hospitals. Both surveys utilized multistage probability sampling procedures, which enable researchers to generate nationally representative estimates. Between 1993 and 2003, annual participation rates among physicians selected for NAMCS averaged 70%, while the participation rate of selected hospitals with outpatient departments was 90% in NHAMCS. Standard encounter forms were completed for a systematic random sample of patient visits during randomly assigned reporting periods. Yearly encounter forms varied slightly between NAMCS and NHAMCS and were revised every 2 y. We based this study on variables common to NAMCS and NHAMCS over time, including patient demographics, visit characteristics, reasons for visit (up to three), diagnoses (up to three), and new and continuing medications (up to five in 1993–1994, six in 1995–2002, and eight in 2003). Item nonresponse rates were mostly 5% or less in both surveys for all years.

### Patients

#### CHD risk categorization

We defined four mutually exclusive categories of CVD risk based on the presence of specific diagnoses and risk factors. The presence of CHD, myocardial infarction, stroke or transient ischemic attack, peripheral vascular disease, claudication, or angina defined high CVD risk. In the absence of known CVD, visits by patients with DM who were older than 40 y or had additional risk factors (i.e., hypertension, smoking, dyslipidemia, and/or albuminuria) were defined as intermediate risk. The remaining patients were defined in a second intermediate risk category if they met either of the following criteria: (1) Two or more major CVD risk factors (i.e., hypertension, smoking, and/or dyslipidemia) among men age 45–54 and women age 55–64; or (2) One or more risk factors among men older than 55 and women older than 65. Patient visits ineligible for any of the former three categories were considered low risk. The absence of data elements such as family history of premature CHD or levels of high-density lipoprotein cholesterol precluded more accurate risk stratification according to the Framingham risk scoring [31].

#### Patient visit characteristics

We included the following patient visit characteristics: patient age, gender, race/ethnicity, health care insurance status, visit status, US census region, metropolitan area status, and physician specialty. Health care insurance was classified as private/commercial insurance, public insurance (i.e., Medicare and Medicaid), and other insurance (e.g., workers' compensation and self-pay). Visit status distinguished first-time visits from return visits to a physician's practice. Physician specialty was available only from NAMCS, which contributed more than 90% of the total weighted visits for each of the study years. We categorized physician specialties into cardiology, internal medicine, general and family practice, and a category encompassing all others.

### Measures

Our primary analytic goals were to assess the probability of aspirin use by CVD risk and its relationship to patient visit characteristics. The probability of aspirin use was defined as the proportion of non-contraindicated patient visits in which aspirin or a therapeutically equivalent medication was reported as a new or continuing medication. Measuring the probability of use by CVD risk provided a means to estimate the gaps between current practice and evidence-based medicine regarding aspirin therapy. We defined aspirin therapy as reported use of generic or brand name forms of aspirin, clopidogrel, ticlopidine, or non-narcotic combination analgesics containing aspirin. The number of patient visits in which clopidogrel or ticlopidine was reported is too small to allow their use over time being tracked separately. Oral anticoagulants are not considered aspirin equivalents and are not recommended for the primary or secondary prevention of CVD in a vast majority of patients. Moreover, judging the appropriateness of using or avoiding aspirin for someone who is already on anticoagulant therapy required more clinical detail than our data sources can provide. Therefore, we felt it was appropriate to exclude patients on anticoagulant therapy. We were unable to assess patients' use of over-the-counter aspirin if it was not reported on the encounter form. We excluded visits by patients younger than 21 y and those with bleeding tendency, gastrointestinal bleeding, anticoagulant therapy, or clinically active hepatic disease.

### Statistical Analyses

Statistical analyses accounting for sampling weights and the complex sample designs of NAMCS and NHAMCS were performed using SAS for Windows software (SAS Institute, Cary, North Carolina, United States) and SAS-callable SUDAAN software (RTI, Research Triangle Park, North Carolina, United States). The unit of analysis in both surveys was the patient visit. Comparisons of NAMCS and NHAMCS suggested limited differences on key outcome measures. We therefore combined the two surveys to obtain a wider range of outpatient settings and a broader socioeconomic spectrum of patients seeking ambulatory care. Also, to minimize random fluctuations between years, we analyzed data in 2-y groupings, except for 2003, for depicting temporal trends in aspirin use. The SAS SURVEYMEANS procedure was performed, which generated national estimates of the probability of aspirin use by CVD risk and corresponding 99% confidence intervals (CIs). We chose to report 99% CIs in following National Center for Health Statistics analytical guidelines and also as a conservative measure to avoid over-interpretation of the findings. Chi-square tests were performed using PROC CROSSTAB in SUDAAN to examine the association of aspirin use with each patient visit characteristic based on combined 1993–2003 NAMCS and NHAMCS data. The independent effect of each patient visit characteristic on aspirin use after controlling for all other characteristics was assessed with a multivariate logistic regression model using PROC RLOGISTIC in SUDAAN. The model produced adjusted odds ratios and 99% CIs.

## Results

The volume of outpatient visits by patients identified as being at elevated risk for future CVD events, particularly those at intermediate risk, rose markedly over the study period. The number of high-risk patient visits increased by 33% from 44.2 (99% CI, 41.0–47.4) million in 1993–1994 to 58.8 (54.0–63.6) million in 2001–2002. The number of intermediate-risk patient visits in which a diagnosis of DM was noted more than doubled, from 40.5 (37.1–43.9) million to 83.3 (77.4–89.3) million, and for those with multiple risk factors the increase was 57%, from 70.2 (65.7–74.7) million to 110.4 (102.8–118.0) million. The number of low-risk patient visits rose by 23%, from 975.4 (962.6–988.2) million to 1.20 (1.18–1.22) billion. In 2003, the number of patient visits in each of the four risk categories was 29.5 (22.5–36.6) million for high-risk patients, 39.9 (32.0–47.9) million for intermediate-risk patients, 55.8 (45.5–66.2) million for those with multiple risk factors, and 626.9 (537.1–716.7) million for those with low risk.

Trends over time showed improved, though still substantially suboptimal, aspirin use in the high and intermediate risk categories, with sustained improvements seen beginning in 1999–2000 (Figure 1). The probability of aspirin use among patient visits in 1993–1994 was 21.7% (18.8%–24.6%) for the high-risk category, 3.5% (2.0%–5.0%) for the diabetic, intermediate-risk category, and 3.6% (2.6%–4.6%) for the other intermediate-risk category. The probabilities for these three risk categories fluctuated somewhat but remained essentially unchanged through 1999–2000. Increases were observed in 2001–2002 and persisted in 2003. The probability of aspirin use in 2003 was 32.8% (25.2%–40.4%) for the high-risk category, 11.7% (7.8%–15.7%) for the diabetic, intermediate-risk category, and 16.3% (11.4%–21.2%) for the other intermediate-risk category. Aspirin use remained 1%–3% among low-risk patient visits.

**Figure 1:**
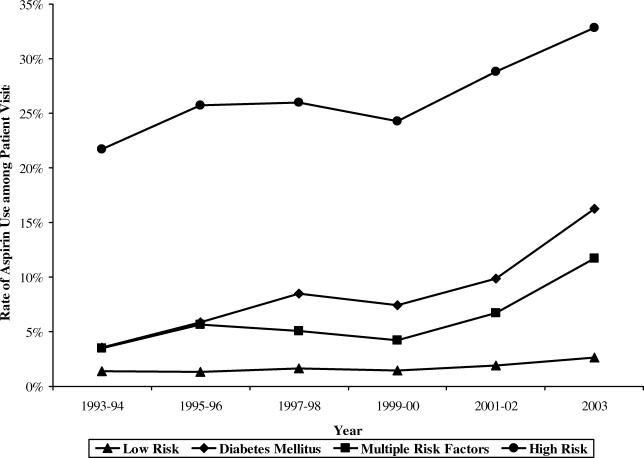
National Trends in Aspirin Use in Patient Visits Defined as Low Risk, Intermediate Risk, DM, or High Risk

To explore the relative priority assigned to aspirin and statins, we examined trends in the co-prescribing of the medications. For this series of analyses, the number of visits by patients with DM was relatively small and were therefore grouped with those with known CVD to compose the high-risk category. Both aspirin and statins were used more frequently when the other therapy was present; however, improvements over time were more evident for statin use among aspirin-treated patient visits than for aspirin use among statin-treated patients. Specifically, the proportion of visits by high-risk patients on aspirin while a statin was used declined modestly from 36.5% (24.9%–48.2%) in 1993–1994 to 25.6% (20.1%–31.1%) in 1999–2000 but then rebounded to 43.9% (35.1%–52.8%) in 2003 (Figure 2). In contrast, statin use among visits by high-risk patients on aspirin grew successively from 11.6% (7.4%–15.7%) to 54.3% (45.7%–63.0%) (Figure 2). Of visits by intermediate-risk patients, the probability of aspirin use when on a statin increased from 6.0% (1.4%–10.6%) in 1993–1994 to 33.8% (21.5%–46.0%) in 2003, while the probability of statin use when on aspirin rose from 8.8% (2.2%–15.3%) to 48.1% (35.2%–61.0%) (Figure 3).

**Figure 2:**
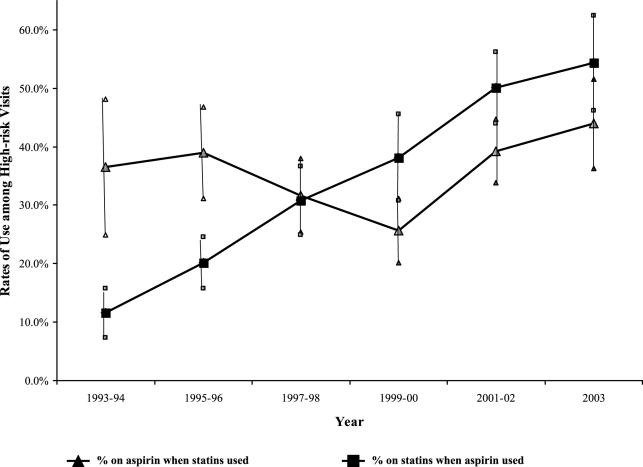
National Trends in Aspirin and Statin Use When the Other Therapy Is Present among High-Risk Patient Visits Vertical bars indicate 99% CIs.

**Figure 3:**
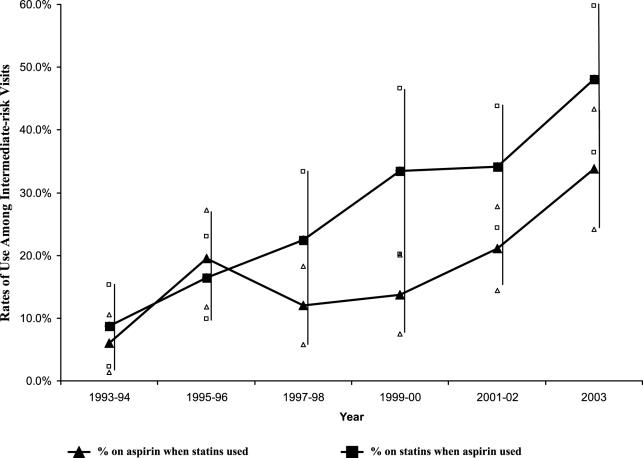
National Trends in Aspirin and Statin Use When the Other Therapy Is Present among Intermediate-Risk Patient Visits Vertical bars indicate 99% CIs.

The association of greater aspirin use with higher CVD risk was confirmed by multivariate logistic regression (Table 1). After adjusting for patient visit characteristics and the number of medications reported, aspirin use was over four time as likely among visits by high-risk patients and approximately two times as likely among visits by patients with multiple risk factors as it was among low-risk patient visits. The odds ratio was marginally significant for the diabetic, intermediate-risk category. The significance of increases in aspirin use over time did not sustain in the multivariate logistic regression. As for patient visit characteristics, lower probability of aspirin use was found among 20- to 44-y-olds (versus those 45 y or older), women (versus men), visits to noncardiologists (versus visits to cardiologists), return visits (versus first-time visits), and visits to private physician offices (versus visits to hospital outpatient departments). Finally, the probability of aspirin use was positively associated with the number of medications reported (odds ratio, 1.71; 99% CI, 1.65–1.76).

**Table 1:**
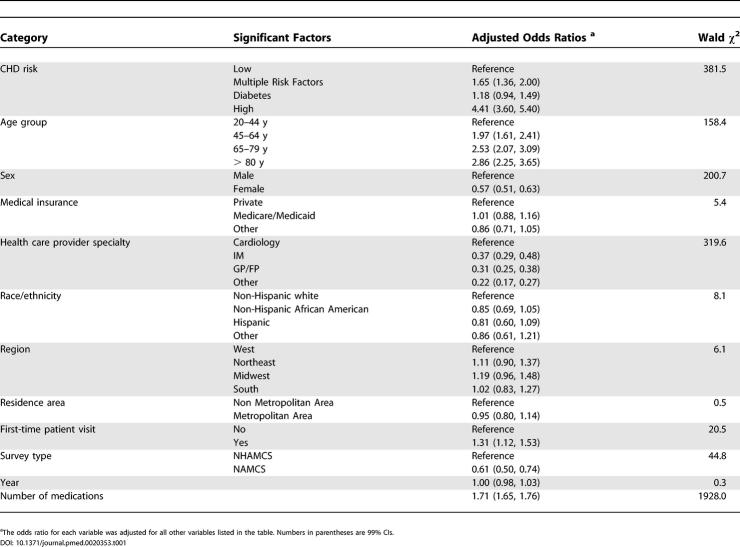
Predictors of Aspirin Use With Combined 1995–2002 NAMCS and NHAMCS Data

## Discussion

This study documents national trends in the probability of aspirin use by CVD risk category among patient visits to office-based physicians and hospital outpatient departments. Some improvements were observed over time in the use of aspirin for both the secondary and primary prevention of CVD. However, the magnitude of those improvements is minimal relative to the substantial gaps between clinical practice and evidence-based recommendations. The gaps observed with secondary prevention are particularly concerning, given the existence of conclusive clinical evidence and unequivocal practice guidelines. The use of aspirin among primary prevention patients, including those with diabetes, also appears to be suboptimal, but additionally may reflect uncertainty about the evidence. Our analysis also suggests that despite aspirin's more favorable cost-effectiveness, statins have been prioritized ahead of aspirin as therapy for reducing CVD risk.

While ample evidence attests to the underuse of aspirin in reducing risks of CVD, this study uniquely provides an 11-y trajectory of aspirin use in US outpatient settings and reveals that improvements have been at best modest. The magnitude of improvements seems particularly small in the context of often-repeated national guidelines and abundant clinical evidence supporting aspirin use for the prevention of CVD, particularly in patients with known CVD. Even in 2003, aspirin use was reported in only one-third of the visits by patients having CVD, which points to widespread under-appreciation of aspirin as an efficacious and cost-effective secondary prevention therapy. The usage was 12% among visits by diabetics, a group at increased cardiovascular risk. This was lower than the 16% found among visits by patients with multiple major cardiovascular risk factors for whom evidence supporting prophylactic aspirin therapy is less definitive. The continued increases in aspirin use since 1999–2000 may reflect heightened awareness of the benefits of aspirin in reducing cardiovascular morbidity and mortality, mediated through intensified dissemination of national guidelines and clinical trial findings [2,9–11,32]. We did not find evidence of aspirin overuse in low-risk patients.

Compared with clinical practice in Europe [22,23], our study results add support to the observation that underuse of aspirin is more problematic in the US. The genesis of this gap is likely multifactorial and open to postulation. For instance, US physicians may face greater pressure than their European colleagues to prescribe newer medications as a result of less-restrictive regulations on drug advertising. Also, direct-to-consumer advertising has been shown to change patient-physician relationships and physician prescribing behavior.

The widespread aspirin underutilization could be partly due to uncertainties in risk assessment. Health care providers show little consistency as to how much risk of excess bleeding is acceptable, which may partly account for the variability in aspirin prescribing [33]. Data indicate that aspirin use is linked to approximately 2.5%–4.5% of the annual upper gastrointestinal events (symptomatic ulcers) and 1%–1.5% of serious complications, such as severe bleeding, perforation, and obstruction [34]. These risk estimates should be evaluated in the context of average reductions of 15−40% in cardiovascular events when aspirin is used as a preventive therapy [3,10,11,14,35]. Accurate risk assessment can be difficult at the individual patient level, especially when discrepancies arise between verbal and written medical history information [36]. Aspirin resistance may also limit the rates of aspirin use. However, the frequency of aspirin resistance is less well known and may range from 5% to 60% [37]. In some patients it may be dose related. Lee et al. [38] indicate that even a low-dose aspirin of 100 mg or less may increase aspirin resistance in patients with coronary artery disease.

Past research also suggests that physicians may assign lower priority to aspirin therapy than to other cardiovascular risk-lowering therapies [25,26,36], and our evaluations of the co-prescription of aspirin and statins support this assessment. We found that aspirin and statin use was significantly higher when the other therapy was present; however, the incremental use became progressively greater for statins over time. Beginning in 1997–1998, statin use in the presence of aspirin transcended aspirin use in the presence of statins for both the high- and intermediate-risk categories, and the gaps remained wide through 2003. These results suggest that even though statins themselves may be underused, aspirin is given even lower priority for lowering cardiovascular risk. These findings are intriguing because both therapies reduce cardiovascular risk by similar magnitudes but differ vastly in cost; statins are prioritized despite the far greater cost-effectiveness of aspirin [39–41]. Also, secondary analyses of clinical trial data indicate that aspirin and statins used in combination may be more effective at reducing the relative risk of CVD events than when used alone [42].

Statins are newer and more intensely advertised than aspirin, which may partly explain the preferential use of these drugs. Lipid-lowering medications already ranked the fifth most promoted drug class in the US in 1998 [43]. Statins are proven effective for both the primary and secondary prevention of CVD, whereas the effectiveness of aspirin in primary prevention is less certain. Also, while they are increasingly used as a prophylactic treatment, statins are still most commonly prescribed to people with hyperlipidemia. In contrast, use of aspirin is not specific to any risk factor in the prevention of CVD and therefore may be neglected by many physicians who are trained to perform in an overly acute-care-centered health care system. In addition, statins may be perceived to have a more favorable side-effect profile than aspirin, which has been shown to increase the risk of severe gastrointestinal and cerebral hemorrhage [34]. Finally, our comparison of aspirin and statin use is confounded by the likelihood of underreporting of over-the-counter aspirin use by participating physicians and clinical staff.

In agreement with previous findings, lower aspirin use is associated with female gender, younger age, noncardiologist care, and care in the private office setting [15,16,27,28,44,45]. The appropriateness of prophylactic aspirin therapy among women, particularly those under 65 y of age, is yet to be determined in light of the new evidence from the Women's Health Study [7]. However, variations of aspirin use by physician specialty and type of health care setting raise questions about equity in the process of care. As a result of high penetration of managed care, patients are increasingly less likely to see a specialist such as cardiologist, unless referred by their primary care provider [45]. Primary care providers, including those who practice in private offices, are expected to adhere more diligently to practice guidelines in this area that was previously the domain of specialists.

Our findings should be interpreted in the context of several limitations of the data sources used. Both NAMCS and NHAMCS are designed to produce national estimates on the basis of patient visits, and they provided no way to link multiple visits by the same patient. The per-patient visit nature of our analysis may lead to overestimation of aspirin use, particularly for high-risk patients, due to more frequent visits by sicker patients and indiscriminate reporting of sporadic and long-term use of aspirin. Individuals who have visited an ambulatory care facility may differ from those who fail to do so or do so less frequently. However, observed aspirin use may underestimate actual administration due to its low-cost, over-the-counter availability, although participating physicians and clinical staff are instructed to record nonprescription medication. In an attempt to indirectly gauge the potential of underreporting of aspirin use, Stafford [16] studied the reporting of multivitamin use during pregnancy and nonprescription analgesic use for osteoarthritis, and concluded that these surveys capture a reasonably substantial proportion of nonprescription medication use. By limiting the number of medications reported to six or fewer, some medications, particularly those perceived as less critical for the treatment of primary diagnoses, may not be reported. When we compared patient visits in which the maximum number of medications were reported with visits in which fewer were reported, we found no differences in the likelihood of aspirin use. If aspirin is, in fact, under-reported, less clinical attention and priority may be given to aspirin use compared to other therapies.

While these data limitations present certain difficulties in interpreting the absolute usage of aspirin, they should have limited impact on our trend analysis. The extent of under-reporting may have attenuated over time due to increased awareness of its effectiveness in cardiovascular risk reduction, which could partly explain the increasing trends in use that we observed. We have no reason to believe that under-reporting varies so substantially by patient visit characteristics that it could have confounded our multivariate logistic analysis.

In conclusion, improvements in aspirin use for reducing risks of CVD among US outpatients are at best modest, and substantial treatment gaps persist, particularly in secondary prevention, for which definitive evidence of benefits is available. Aspirin is more underused than statins despite its more favorable cost-effectiveness. Marked changes in clinical practice are unlikely to occur unless more aggressive, innovative means are implemented to enhance health care provider and patient adherence to consensus guidelines on aspirin therapy to prevent CVD events. In particular, targeted interventions may be warranted in patient subpopulations in which aspirin use is lower than average, including women, young adults, and ethnic minorities. Targeted continuing medical education for primary care providers especially in solo or small-group practices, may introduce greater consistency into practice by specialty and practice setting.

**Patient Summary**

**Background**

Aspirin is known to be effective in lessening the chance of heart attack, stroke, or other cardiovascular diseases that may occur when blood vessels are blocked by blood clots. Therefore, guidelines recommend that certain groups of people take aspirin regularly either to prevent such clots forming in the first place, or after such a clot has formed to prevent further clots. However, aspirin may increase the chance of bleeding in some people; hence it is important that the benefits of taking aspirin are balanced against possible side effects.

**Why Was This Study Done?**

The researchers wanted to investigate temporal patterns of aspirin use among patients who would potentially benefit from taking it, and ask whether there were any particular reasons—for either patients or their health care providers—that influenced such use.

**What Did the Researchers Do and Find?**

They used data over 11 years from two nationwide surveys in the US that study prescribing patterns in outpatients. Some improvements were observed between 1993 and 2003 in the use of aspirin among patients with known CVD and those without. However, the magnitude of those improvements is minimal relative to the substantial gaps between clinical practice and evidence-based recommendations. From 1997 to 1998 onward, statins were used more frequently compared with aspirin as prophylactic therapy for reducing cardiovascular disease risk. Greater aspirin use was seen most frequently in people of advanced age, who were male, who were being cared for by cardiologists (rather than general physicians or other specialists), and who were being seen in hospital outpatient departments (rather than private practices).

**What Do These Findings Mean?**

Although there is very good evidence that aspirin is particularly useful when given after a cardiovascular event—so-called secondary prevention—there were only modest increases in the use of aspirin in this period. Aspirin is less frequently used than statins, despite its greater cost-effectiveness. Innovative interventions are needed to enhance patients' and health care providers' understanding of and adherence to the guidelines that have been developed on reducing the risk of cardiovascular disease.

**Where Can I Get More Information Online?**

MedlinePlus has information on aspirin and related drugs:

http://www.nlm.nih.gov/medlineplus/druginfo/uspdi/202515.html

Omni is a UK-based free catalog of hand-selected and evaluated Internet resources in health and medicine, including a page of links on aspirin:

http://omni.ac.uk/browse/mesh/D001241.html

## Abbreviations

DM: diabetes mellitus
CHD: coronary heart disease
CI: confidence interval
CVD: cardiovascular disease
NAMCS: National Ambulatory Medical Care Survey
NHAMCS: National Hospital Ambulatory Medical Care Survey

## References

[pmed-0020353-b001] Berg AO, Atkins D (2002). Aspirin for the primary prevention of cardiovascular events. US Preventive Services Task Force. Ann Intern Med.

[pmed-0020353-b002] Pearson TA, Blair SN, Daniels SR, Eckel RH, Fair JM (2002). AHA guidelines for primary prevention of cardiovascular disease and stroke: 2002 update: Consensus panel guide to comprehensive risk reduction for adult patients without coronary or other atherosclerotic vascular diseases. American Heart Association Science Advisory and Coordinating Committee. Circulation.

[pmed-0020353-b003] Hansson L, Zanchetti A, Carruthers S, Dahlof B, Elmfeldt D (1998). Effects of intensive blood-pressure lowering and low-dose aspirin in patients with hypertension: principal results of the Hypertension Optimal Treatment (HOT) randomized trial. HOT Study Group. Lancet.

[pmed-0020353-b004] Steering Committee for the Physician's Health Study Research Group (1989). Final report on the aspirin component of the ongoing Physician's Health Study. The Steering Committee for the Physician's Health Study Research Group. N Engl J Med.

[pmed-0020353-b005] Medical Research Council's General Practice Research Framework (1998). Thrombosis prevention trial: Randomized trial of low-intensity oral anticoagulation with warfarin and low-dose aspirin in the primary prevention of ischaemic heart disease in men at increased risk. The Medical Research Council's General Practice Research Framework. Lancet.

[pmed-0020353-b006] Collaborative Group of the Primary Prevention Project (2001). Low-dose aspirin and vitamin E in people at cardiovascular risk: A randomized trial in general practice. Collaborative Group of the Primary Prevention Project. Lancet.

[pmed-0020353-b007] Ridker PM, Cook NR, Lee IM, Gordon D, Gaziano JM, Manson JE (2005). A randomized trial of low-dose aspirin in the primary prevention of cardiovascular disease in women. N Engl J Med.

[pmed-0020353-b008] ISIS-2 Collaborative Group (1988). Randomized trial of intravenous streptokinase, oral aspirin, both, or neither among 17,187 cases of suspected acute myocardial infarction: ISIS-2. ISIS-2: Second International Study of Infarct Survival, Collaborative Group. Lancet.

[pmed-0020353-b009] Anti-Platelet Trialists' Collaboration (2002). Collaborative meta-analysis of randomized trials of anti platelet therapy for prevention of death, myocardial infarction, and stroke in high risk patients: Anti-Platelet Trialists' Collaboration. Br Med J.

[pmed-0020353-b010] Weisman SM, Graham DY (2002). Evaluation of the benefits and risks of low-dose aspirin in the secondary prevention of cardiovascular and cerebrovascular events. Arch Intern Med.

[pmed-0020353-b011] Chen Z, Sandercock P, Pan H, Counsell C, Collins R (2000). Indications for early aspirin use in acute ischemic stroke. A combined analysis of 40,000 randomized patients from the Chinese Acute Stroke Trial and the International Stroke Trial. Stroke.

[pmed-0020353-b012] Harrington RA, Becker RC, Ezekowitz M, Meade TW, O'Connor CM (2004). Antithrombotic therapy for coronary artery disease: The Seventh ACCP Conference on Antithrombotic and Thrombolytic Therapy. Chest.

[pmed-0020353-b013] Albers GW, Amarenco P, Easton JD, Sacco RL, Teal P (2004). Antithrombotic and thrombolytic therapy for ischemic stroke: The Seventh ACCP Conference on Antithrombotic and Thrombolytic Therapy. Chest.

[pmed-0020353-b014] American Diabetes Association (2004). Aspirin therapy in diabetes. Diabetes Care.

[pmed-0020353-b015] Stafford RS, Radley DC (2003). The underutilization of cardiac medications of proven benefit, 1990 to 2002. J Am Coll Cardiol.

[pmed-0020353-b016] Stafford RS (2000). Aspirin use is low among United States outpatients with coronary artery disease. Circulation.

[pmed-0020353-b017] Rolka DB, Fagot-Campagna A, Narayan KM (2001). Aspirin use among adults with diabetes. Estimates from the Third National Health and Nutrition Examination Survey. Diabetes Care.

[pmed-0020353-b018] Kramer JM, Newby LK, Chang WC, Simes RJ, Van de Werf F (2003). International variation in the use of evidence based medicines for acute coronary syndromes. Eur Heart J.

[pmed-0020353-b019] Venturini F, Romero M, Tognoni G (1999). Patterns of practice for acute myocardial infarction in a population from ten countries. Eur J Clin Pharmacol.

[pmed-0020353-b020] O'Connor GT, Quinton HB, Traven ND, Ramunno LD, Dodds TA (1999). Geographic variation in the treatment of acute myocardial infarction: The Cooperative Cardiovascular Project. JAMA.

[pmed-0020353-b021] Bennett KE, Williams D, Feely J (2003). Under-prescribing of cardiovascular therapies for diabetes in primary care. Eur J Clin Pharmacol.

[pmed-0020353-b022] EUROASPIRE II (2001). Lifestyle and risk factor management and use of drug therapies in coronary patients from 15 countries: Principal results from EUROASPIRE II Euro Heart Survey Programme. EUROASPIRE II Study Group. Eur Heart J.

[pmed-0020353-b023] Steffenino G, Galliasso M, Gastaldi C, Ricca N, Mangiacotti B (2003). Nurses' observational study on the practice of secondary prevention in a cardiovascular department. Ital Heart J.

[pmed-0020353-b024] Avezum A, Makdisse M, Spencer F, Gore JM, Fox KAA (2005). Impact of age on management and outcome of acute coronary syndrome: observations from the Global Registry of Acute Coronary Events (GRACE). Am Heart J.

[pmed-0020353-b025] Meigs JB, Stafford RS (2000). Cardiovascular disease prevention practices by U.S. physicians for patients with diabetes. J Gen Intern Med.

[pmed-0020353-b026] Califf RM, DeLong ER, Ostbye T, Muhlbaier LH, Chen A (2002). Underuse of aspirin in a referral population with documented coronary artery disease. Am J Cardiol.

[pmed-0020353-b027] Ganz DA, Lamas GA, Orav EJ, Goldman L, Gutierrez PR (1999). Age related differences in management of heart disease: A study of cardiac medication use in an older cohort. Pacemaker Selection in the Elderly (PASE) Investigators. J Am Geriat Soc.

[pmed-0020353-b028] Krumholz HM, Radford MJ, Ellerbeck EF, Hennen J, Meehan TP (1995). Aspirin in the treatment of acute myocardial infarction in elderly Medicare beneficiaries. Patterns of use and outcomes. Circulation.

[pmed-0020353-b029] Zell ER, McCaig LF, Kupronis BA, Besser RE, Schuchat A (2000). A comparison of the National Disease and Therapeutic Index and the National Ambulatory Medical Care Survey to evaluate antibiotic usage. Proceedings of the survey research methods section, American Statistical Association.

[pmed-0020353-b030] Gilchrist VJ, Stange KC, Flocke SA, McCord G, Bourguet CC (2004). A comparison of the National Ambulatory Medical Care Survey (NAMCS) measurement approach with direct observation of outpatient visits. Medical Care.

[pmed-0020353-b031] Wilson PWF, D'Agostino RB, Levy D, Belanger AM, Silbershatz H (1998). Prediction of coronary heart disease using risk factor categories. Circulation.

[pmed-0020353-b032] Colwell JA (2001). Aspirin therapy in diabetes is underutilized. Diabetes Care.

[pmed-0020353-b033] Devereaux PJ, Anderson DR, Gardner MJ, Putnam W, Flowerdew GJ (2001). Differences between perspectives of physicians and patients on anticoagulation in patients with atrial fibrillation: Observational study. Br Med J.

[pmed-0020353-b034] Laine L (2002). The gastrointestinal effects of nonselective NSAIDs and COX-2-selective inhibitors. Semin Arthritis Rheum.

[pmed-0020353-b035] ETDRS (1992). Aspirin effects on mortality and morbidity in patients with diabetes mellitus. Early Treatment Diabetic Retinopathy Study report 14: ETDRS Investigators. JAMA.

[pmed-0020353-b036] Short D, Frischer M, Bashford J, Ashcroft D (2003). Why are eligible patients not prescribed aspirin in primary care? A qualitative study indicating measures for improvement. BMC Fam Pract.

[pmed-0020353-b037] Martin CP, Talbert RL (2005). Aspirin resistance: an evaluation of current evidence and measurement methods. Pharmacotherapy.

[pmed-0020353-b038] Lee PY, Chen WH, Ng W, Cheng X, Kwok JY, Tse HF, Lau CP (2005). Low-dose aspirin increases resistance in patients with coronary artery disease. Am J Med.

[pmed-0020353-b039] Marshall T (2003). Coronary heart disease prevention: Insights from modelling incremental cost effectiveness. Br Med J.

[pmed-0020353-b040] Drummond A, Kwok S, Morgan J, Durrington PN (2001). Costs of aspirin and statins in general practice. QJM.

[pmed-0020353-b041] Probstfield JF (2003). How cost-effective are new preventive strategies for cardiovascular disease?. Am J Cardiol.

[pmed-0020353-b042] Hennekens CH, Sacks FM, Tonkin A, Jukema JW, Byington RP (2004). Additive benefits of pravastatin and aspirin to decrease risks of cardiovascular disease. Arch Intern Med.

[pmed-0020353-b043] Ma J, Stafford RS, Cockburn IM, Finkelstein SN (2003). A statistical analysis of the magnitude and composition of drug promotion in the United States in 1998. Clin Ther.

[pmed-0020353-b044] Frances CD, Go AS, Dauterman KW, Deosaransingh K, Jung DL (1999). Outcome following acute myocardial infarction: Are differences among physician specialties the result of quality of care or case mix?. Arch Intern Med.

[pmed-0020353-b045] Akosah KO, Larson DE, Brown WM, Paul K, Schaper A (2003). Using a systemwide care path to enhance compliance with guidelines for acute myocardial infarction. Jt Comm J Qual Saf.

